# Self-reported traffic-related air pollution and respiratory symptoms among adults in an area with modest levels of traffic

**DOI:** 10.1371/journal.pone.0226221

**Published:** 2019-12-12

**Authors:** Marit Nøst Hegseth, Bente Margaret Oftedal, Anje Christina Höper, Anna Louise Aminoff, Marte Renate Thomassen, Martin Veel Svendsen, Anne Kristin Møller Fell

**Affiliations:** 1 Department of Occupational and Environmental Medicine, University Hospital of North Norway, Tromsø, Norway; 2 Institute of Community Medicine, UiT- The Arctic University of Norway, Tromsø, Norway; 3 Department of Air Pollution and Noise, Norwegian Institute of Public Health, Oslo, Norway; 4 Department of Occupational and Environmental Medicine, Telemark Hospital, Skien, Norway; Ruhr University Bochum, GERMANY

## Abstract

Health effects of traffic-related air pollution (TRAP) concentrations in densely populated areas are previously described. However, there is still a lack of knowledge of the health effects of moderate TRAP levels. The aim of the current study, a population-based survey including 16 099 adults (response rate 33%), was to assess the relationship between TRAP estimates and respiratory symptoms in an area with modest levels of traffic; Telemark County, Norway. Respondents reported respiratory symptoms the past 12 months and two TRAP exposure estimates: *amount of traffic outside their bedroom window* and *time spent by foot daily along a moderate to heavy traffic road*. Females reported on average more symptoms than males. Significant relationships between *traffic outside their bedroom window* and number of symptoms were only found among females, with the strongest associations among female occasional smokers (incidence rate ratio [IRR], 1.75, 95% confidence interval (CI) [1.16–2.62] for moderate or heavy traffic compared to no traffic). Significant relationship between *time spent daily by foot along a moderate to heavy traffic road* and number of symptoms was found among male daily smokers (IRR 1.09, 95% CI [1.04–1.15] per hour increase). Associations between *traffic outside bedroom window* and each respiratory symptom were found. Significant associations were primarily detected among females, both among smokers and non-smokers. Significant associations between *time spent by foot daily along a moderate to heavy traffic road* (per hour) and *nocturnal dyspnoea* (odds ratio (OR) 1.20, 95% CI [1.05–1.38]), *nocturnal chest tightness* (OR 1.13 [1.00–1.28]) and *wheezing* (OR 1.14 [1.02–1.29]) were found among daily smokers, primarily men. Overall, we found significant associations between self-reported TRAP exposures and respiratory symptoms. Differences between genders and smoking status were identified. The findings indicate an association between TRAP and respiratory symptoms even in populations exposed to modest levels of TRAP.

## Introduction

Traffic-related air pollution (TRAP) is one of the main sources of urban air pollution[1–4]. TRAP consists of primarily gaseous emissions, including nitrogen oxides (NOx) and carbon monoxide, as well as particulate matter (PM). TRAP PM originates from combustion and non-combustion sources such as sanding and salting of the roads during winter, road dust and tire wear. Additionally, secondary pollutants such as ozone, nitrates and organic aerosol may form. PM, including ultrafine particles (UFP), may carry substances such as polycyclic aromatic hydrocarbons, transition metals and environmentally persistent free radicals as well as immunogenic substances such as pollen or fungal spores.

TRAP is a known risk factor for respiratory diseases such as chronic obstructive pulmonary disease (COPD) and asthma[5–7]. PM is the TRAP component that is considered the main cause of respiratory morbidity [8], although NOx have also been associated with both new onset of COPD and asthma[6, 9]. Several respiratory symptoms including wheezing, chronic bronchitis, chronic phlegm and reduced lung function have been reported in populations living in areas with documented TRAP[10–15]. Consistent associations between TRAP at the home address and respiratory morbidity have been revealed for children and adolescents [3, 16–21], and similar findings have been observed in adult populations[9–13, 22, 23].

The relationship between TRAP exposure and respiratory morbidity has been studied worldwide[10, 12, 23–25], particularly in European urban areas[22, 26]. Although some investigations have included populations in less densely populated areas, such as smaller cities in Scandinavia[9, 27] or rural areas in Switzerland[22], most studies have focussed on densely populated metropolitan areas[11, 13, 26, 28]. Hence, there is still a lack of knowledge of the associations between moderate TRAP levels and health effects.

Rural and urban areas differ significantly in TRAP concentrations, often with concentrations twice as high in urban than rural areas, and 2–3 times the urban levels close to busy traffic ores[4]. This is reflected in the increased respiratory morbidity observed among those who live close to high traffic roads in urban areas[12–14, 17, 22, 29]. The highest concentrations of TRAP constituents such as NO_2_ and UFP have been found within 30 m of the road, decreasing exponentially to background levels approximately 300 m away[30].

Most of Telemark County consists of rural areas, and even the urban area in Grenland is moderately trafficked compared to other European cities[31]. Telemark County has approximately 100 km of roads with annual average daily traffic (AADT) >10 000 vehicles per day, and the Grenland area is the most densely populated area of Telemark County and the area with the busiest roads. 173 000 persons live in Telemark County, and approximately 120 000 of these live in the Grenland area, of which 90 000 live in the neighbouring cities Porsgrunn and Skien[32]. The AADT of the main road into the Grenland area was 18 000 vehicles per day in 2017[31]. The other roads are less busy (<10 000 vehicles per day). In this regard, even the most busy roads in the most densely populated areas of Telemark are moderately trafficked in a global, urban context[30, 33–36].

Generally, in all urban areas in Norway, the annual PM_2,5_, PM_10_ and NO_2_ measurements are within the WHO[37] and national[38] air quality guidelines. The highest concentrations of PM_2,5_, PM_10_ and NO_2_ are measured near major traffic ores in Oslo, with annual means in 2017 of 9, 22 and 41 ug/m3, respectively [39]. Measurements from the urban areas of Telemark county show lower levels of annual PM_2,5_ and PM_10_; 7 and 16 μg/m^3^, respectively[40]. Compared to other areas globally, for example cities like Cairo (PM_2,5_ 117 μg/m^3^ and PM_10_ 284 μg/m^3)^, Dehli (PM_2,5_ 143 μg/m^3^ and PM_10_ 292 μg/m^3^) and Beijing (PM_2,5_ 73 μg/m^3^ and PM_10_ 92 μg/m^3^)[41], the annual mean concentrations of PM_2,5_ and PM_10_ in Norway, including Telemark County, are modest.

Nevertheless, an elevated use of asthma medication, prevalence of asthma and respiratory symptoms have been observed in Telemark County compared to the rest of Norway and Scandinavia[42]. This topic has been addressed in several previous publications from the Telemark study[42, 43]; occupational exposure, particularly for workers in agriculture/fisheries and craft related/trade workers, was shown to be a contributing factor, but does not offer the full explanation for the over-representation of asthma and respiratory symptoms. Environmental pollution such as indoor and outdoor allergens, second hand smoking, mould or other water damage related irritants or TRAP, may represent additional risk factors explaining this observation, especially in vulnerable sub-populations. Only a few large population-based studies of associations between modest air pollution levels and respiratory illness exist, and new findings may contribute valuable information for regulatory purposes.

The objective of the study was to assess the relationship between TRAP estimates close by roads and reported respiratory symptoms in adults in an area with modest TRAP levels—Telemark County, Norway. The relationship was also investigated in vulnerable sub-groups such as gender and smoking status.

## Materials and methods

### Study participants

The data presented in this paper originates from a cross-sectional population-based survey that was the first wave of data collection in the Telemark study. The Telemark study is described in previous publications[42–44]; but briefly, the study subjects (50 000) were randomly sampled from the total of ∼170 000 inhabitants of Telemark County in the southeastern part of Norway. The survey was carried out from February to August 2013. During this period the participants received a postal questionnaire, and two reminders. The participants were 16–50 years of age and were selected from the national population registry, which contained the personal identification number, date of birth and residential address for each person. The questionnaire was sent by mail to 48 142 eligible subjects (traceable through a postal address).

All information was collected with the participants’ informed and written consent. Participants were also informed that they at any time have the opportunity to withdraw from the study without giving a reason. In accordance with Norwegian legislation the participants between 16 and 18 do not need consent from parents or guardians. The study protocol was conducted with the written approval of the Regional Committee for Medical and Health Research Ethics in Norway in 2012 (2012/1665/REK Sør-øst D).

### Study design

#### Outcomes

The questionnaire was based on the West Sweden Asthma Study questionnaire and the European Community Respiratory Health Survey questionnaire[45]. The presence of respiratory symptoms in the past 12 months was based on an affirmative response to the respective questions regarding *wheezing*: ‘Have you had whistling or wheezing in the chest at any time during the past 12 months?’; *nocturnal chest tightness*: ‘Have you woken up with a feeling of tightness in your chest at any time during the past 12 months?’; *nocturnal dyspnoea*: ‘Have you woken up with dyspnoea at any time during the past 12 months?’; *nocturnal coughing*: “Have you woken up with attacks of coughing at any time during the past 12 months?” and *asthma*: ‘Have you had an asthma attack during the last 12 months?’. Additionally, a sum score for the combined respiratory symptoms was generated for each respondent, where an affirmative answer to one or more of the above described questions was summarised. This generated a value between 0 and 5, describing the symptom burden for each respondent.

#### Exposure assessment

Exposure to TRAP close by roads was assessed through two questions in the questionnaire. First: ‘Is your bedroom window near a street (less than 20 m)?’ with response alternatives: Yes, heavy traffic (HT)–Yes, moderate traffic (MT)–Yes, little traffic (LT)–No (N). The other question was: ‘How much time do you usually spend travelling by foot along a moderate- to heavy-trafficked road in the course of a normal day?’ with the respondents estimating the time spent along a busy road in minutes.

### Statistics

Binary logistic regression was used to estimate the associations between TRAP estimates close by roads (*traffic outside bedroom window* and *time by foot daily along a moderate- to heavy-trafficked road*) and the specific respiratory symptoms. The results are presented as odds ratios (OR) with 95% confidence intervals (CI). The responses to the question *time by foot along a moderate- to heavy- trafficked road* were recalculated from minutes to hours prior to statistical analyses to make the interpretations of the regression analysis results more relevant. Analyses were run both including and excluding the 13 individuals reporting more than 10 hours daily along a busy road, but there were no significant differences in the results, and all individuals were kept in the analyses. Given the distribution and nature of the *number of respiratory symptoms* variable, associations between TRAP estimates and *number of respiratory symptoms* were analysed using negative binomial regression, presented as incidence rate ratios (IRR) with 95% CI for unadjusted and adjusted models. The estimates were adjusted for gender, age, variables indicating socioeconomic status (marital status, educational level, employment status, type of housing) and other relevant exposure estimates (smoking status, number of pack-years, number of cigarettes per day, second-hand smoking, exposure to gas/dust at work, presence of dampness, mould or water damage in the house during the last 10 years and whether they lived in or outside of the Grenland area). There were considerable numbers of missing responses in the variables *pack years* and *number of cigarettes per day*, 10 496 and 10 344, respectively. To be able to include these variables in the model without these individuals being discarded in the analyses, the missing values were replaced by the average number of pack-years and the average number of cigarettes per day, respectively, calculated for their respective smoking status group. Previous findings suggest there may be gender differences with regards to reporting asthma symptoms related to TRAP[9], and to identify gender as a possible sensitive sub group in the current study, stratification was performed. Interactions between exposure variables, gender and smoking status were explored by adding generated product terms with each exposure. The statistical significance of the interactions was evaluated to judge whether stratification by gender and smoking status should be done. Statistical significance level was set to 10% for the interaction analyses. Due to small sample sizes in some of the strata, the TRAP estimate *traffic outside bedroom window* and categories *moderate traffic (MT)* and *heavy traffic (HT)* were combined in the category *moderate or heavy traffic (MHT)*. Gender differences in mean *number of respiratory symptoms* and prevalence of self-reported respiratory symptoms in the last 12 months between female and male respondents were tested using Mann–Whitney U and χ2 tests, respectively.

One of the assumptions made in this study was that all the symptoms included in the questionnaire were related to asthma. Hence, there was expected to be a certain degree of dependence between reporting one or more of these symptoms. Correlation analyses (Spearman) were run between all the five symptoms.

Except for the interactions, the statistical significance level was set to 5% and results reaching significance are marked in bold in the tables. Statistical analyses were performed using IBM SPSS Statistics for Windows (V.24.0.0.2, IBM SPSS, Armonk, New York, USA).

## Results

The response rate of the questionnaire was 33% (n = 16 099). The low study response rate in the Telemark study must be considered. However, non-participation was addressed in a separate study which performed inverse probability weighting to account for non-response bias[42, 44]. In that study, demographic characteristics and respiratory symptoms were reported for the non-responders and possible selection bias assessed. No significant differences were detected for the prevalence of respiratory symptoms (with exception for cough) or asthma between responders and non-responders. We performed analyses with and without weighted data sets but found that the weighting had little effect on the study outcomes. Although the non-response assessments demonstrated that exposure-outcome associations were not affected by non-response, results may not be entirely representative of the initial population.

Population characteristics are shown in Table 1. There were 56% female and 44% male responders, 55% never-smokers and 21% past smokers. Of the smokers, 15% were daily smokers and 9% were occasional smokers. The daily and past smokers had more pack-years and more cigarettes per day than the occasional smokers. *Number of minutes spent by foot daily along a road with moderate to heavy traffic* had 21% missing responses. The range varied from 0 to 16.7 hours (999 minutes), 25th percentile was 0.0 hours (0 minutes), median 0.17 hours (10 minutes) and 75th percentile 0.5 hours (30 minutes). The distribution was right skewed, with most individuals spending no or little time along moderate or busy roads daily, while a minority reported that they spent more than 30 minutes by foot along busy roads. Thirteen individuals responded that they spent more than 10 hours daily along busy roads. The analyses were also run with these individuals excluded, but the results did not change significantly (results not shown). 10% reported *moderate to heavy traffic* and 35% reported *little traffic* outside their bedroom windows.

**Table 1:** Demographics for the study population in the Telemark study.

Demographics	Categories	Total		Female		Male	
		N	%	n	%	n	%
**Age (years)**	16–30	5282	33	2938	33	2344	33
	31–40	4126	26	2373	27	1753	24
	41–50	6691	42	3629	41	3062	43
**Marital Status**	Single	4733	29	2387	27	2346	33
	Married	6398	40	3653	41	2745	38
	Partner	3946	25	2279	26	1667	23
	Divorced/separated	693	4	453	5	240	3
	Widow/widower	56	.3	23	.3	33	.5
	Missing	273	1.7	145	1.6	128	1.8
**What is your highest completed education?**	Elementary school + 1–2 years	2615	16	1360	15	1255	18
	Upper secondary and certificate	6329	39	3089	35	3240	45
	University > = 4	6477	40	4168	47	2309	32
	Other and missing	678	4	323	4	355	5
**Have you been employed the past 12 months?**	No	2702	17	1578	18	1124	16
	Yes	13302	83	7310	82	5992	84
**Smoking categories**	Never	8607	55	4802	55	3805	55
	Past	3272	21	1844	21	1428	21
	Occasionally	1454	9	756	9	698	10
	Daily	2295	15	1263	15	1032	15
	Missing	129	.8	78	.8	58	.8
Number of pack years, median (25–75 percentiles) (Missing = 10496)	Never	0		0		0	
	Past	8,6 (2,9–10,0)		7,5 (2,3–8,7)		8,7 (3,8–12,7)	
	Occasionally	4,8 (0,5–4,8)		4,5 (0,6–4,8)		4,8 (0,45–4,8)	
	Daily	13,0 (6,3–17,3)		12,1 (6,0–16,2)		13,9 (7,0–18,9)	
Number of cigarettes per day, median (25–75 percentiles) (Missing = 10344)	Never	0		0		0	
	Past	11,5 (7,1–15,0)		10,0 (5,0–14,3)		11,5 (10,0–15,0)	
	Occasionally	6,0 (0,7–7,1)		5,9 (0,7–6,0)		6,0 (0,7–10,0)	
	Daily	12,0 (7,1–15,0)		10,0 (7,1–15,0)		13,0 (8,0–15,0)	
**Are you exposed to smoking in your current home?**	Never	14115	88	7871	88	6244	87
	Yes	1745	11	930	10	815	11
	Missing	239	2	139	2	100	1
**What type of house do you live in now?**	Detached house	11181	70	6137	69	5044	71
	Row house	1565	10	923	10	642	9
	Apartment	2879	18	1634	18	1245	18
	Other	325	2	171	2	154	2
**Have you in the course of the last 10 years seen signs of moisture damage, water damage or mold in your home?**	No	13009	81	7145	80	5864	82
	Yes	3090	19	1795	20	1295	18
**Do you live in the Grenland area?**	No	5803	36	3238	36	2565	36
	Yes	10296	64	5702	64	4594	64
**Is your bedroom window near a street (less than 20 m)?**	No	8812	55	4809	54	4003	56
	Little traffic (LT)	5584	35	3104	35	2480	35
	Moderate or heavy traffic (MHT)	1582	10	956	11	626	9
	Missing	121	1	71	1	50	1

Total values in addition to values by gender are given as frequency (n) and percentage (%). Total n = 16 099, response rate 33%.

The mean *number of respiratory symptoms* and prevalence of self-reported respiratory symptoms in the last 12 months among female and male respondents are described in Table 2. Females reported a higher number of respiratory symptoms than men.

**Table 2:** Number of respiratory symptoms Frequency (n) and percentage (%) of responders reporting each respiratory symptom in the last 12 months.

Respiratory symptom, sometime in the last 12 months	Total (n = 16 099)	Female (n = 8940)	Male (n = 7159)
*Number of respiratory symptoms* (mean/std. dev/25-50-75^th^ percentiles)	2.22/3.07/0.0–1.0–3.0	2.36/3.21/0.0–1.0–3.0**	2.05/2.86/0.0–1.0–3.0
	*Yes*, *n (%)*	*Yes*, *n (%)*	*Yes*, *n (%)*
**Asthma attack**	664(4)	453 (5)*	211 (3)
**Nocturnal coughing**	3840 (24)	2581 (29)*	1259 (18)
**Nocturnal dyspnoea**	1179 (7)	676 (8)	503 (7)
**Nocturnal chest tightness**	2333 (14)	1424 (16)	909 (13)
**Wheezing**	3226 (20)	1855 (21)	1371 (19)

* = difference between genders, p< = .0001 (χ2)

** = difference between genders, p< = .05 (Mann-Whitney U)

Values for the total population and by gender are given.

As expected, all reported symptoms were significantly correlated (S1 Table, supporting information). Despite this relationship between the reported symptoms, analyses to assess the relationship between each symptom and the exposure variables were considered valuable.

The relationships between *traffic outside bedroom window*, *time spent by foot daily along a moderate to heavy traffic road* and *number of respiratory symptoms* are shown in Table 3. We found associations in the unadjusted model for both TRAP exposures, which were reduced in the adjusted model, but still statistically significant.

**Table 3:** Unadjusted and adjusted incidence rate ratios (IRR) and 95% confidence intervals for the associations between self-reported traffic outside bedroom window and time spent daily by foot along a moderate to heavy trafficked road with number of respiratory symptoms for the respondents in the Telemark study.

Total n = 16099		Summarized respiratory symptoms last 12 months (*number of respiratory symptoms*)		
		Traffic outside bedroom window		Time spent daily by foot along a moderate to heavy traffic road - 1hour increase
Crude IRR		No	1	**1,08 (1,04–1,12)**
		LT	**1,13 (1,06–1,20)**	
		MHT	**1,48 (1,36–1,62)**	
IRRadj*, total population		No	1	**1,05 (1,01–1,09)**
		LT	**1,09 (1,02–1,16)**	
		MHT	**1,25 (1,13–1,38)**	
IRRadj** Never smokers	Male	No	1	1,02 (0,94–1,10)
		LT	1,14 (0,98–1,33)	
		MHT	1,21 (0,93–1.58)	
	Female	No	1	1,02 (0,93–1,11)
		LT	1,05 (0,93–1,18)	
		MHT	**1,27 (1,06–1,52)**	
IRRadj** Previous smokers	Male	No	1	1,02 (0,93–1,12)
		LT	0,90 (0,71–1,15)	
		MHT	1,25 (0,86–1,84)	
	Female	No	1	1,05 (0,92–1,20)
		LT	1,13 (0,94–1,37)	
		MHT	**1,35 (1,00–1,83)**	
IRRadj** Occasional smokers	Male	No	1	1,11 (0,98–1,27)
		LT	0,85 (0,62–1,15)	
		MHT	1,40 (0,88–2,20)	
	Female	No	1	1,15 (0,85–1,54)
		LT	**1,34 (1,02–1,75)**	
		MHT	**1,75 (1,16–2,62)**	
IRRadj** Daily smokers	Male	No	1	**1,09 (1,04–1,15)**
		LT	0,98 (0,84–1,15)	
		MHT	1,22 (0,97–1,54)	
	Female	No	1	1,04 (0,95–1,14)
		LT	**1,25 (1,06–1,46)**	
		MHT	1,17 (0,93–1,46)	

* Adjusted for gender, age, marital status, educational level, employment status, living in Grenland area, type of housing, dampness/mould or water damage in house in the last 10 years, second-hand smoking, smoking status (never, previous, occasional, daily), number of pack-years, number of cigarettes per day, dust/gas exposure at work, and the other TRAP exposure.

** Adjusted for age, marital status, educational level, employment status, living in Grenland area, type of housing, dampness/mould or water damage in house in the last 10 years, second-hand smoking, number of pack-years, number of cigarettes per day, dust/gas exposure at work, and the other TRAP exposure.

*Results for the total population and strata by gender and smoking status*. *LT = little traffic*. *MHT = moderate or heavy traffic*.

Significant interactions (p<0.10) between smoking status and *traffic outside bedroom window* were revealed for *nocturnal dyspnoea* and *nocturnal chest tightness*. The interaction between gender and *traffic outside bedroom window* was significant (p<0.01) for *asthma attacks*. The interaction between smoking status and *time spent by foot daily along a moderate to heavy trafficked road* was significant for *wheezing* (p<0.05). Finally, significant interactions between smoking status, exposure estimates and gender were revealed when *number of respiratory symptoms* was considered (p<0.05). Because of these findings, stratification by smoking status and gender was performed for all strata. After stratification, significant relationships between estimated traffic outside the bedroom window and reported number of symptoms were only found among females with strongest associations among female occasional smokers. In contrast, the only statistically significant relationship between *time spent daily by foot along a moderate to heavy trafficked road* and reported number of symptoms was found among male daily smokers. Among occasional smokers of both genders, increased associations of similar magnitude were found, although not statistically significant.

Associations between the reported *traffic outside bedroom window* and each of the five different respiratory symptoms are presented by OR, a crude and adjusted model for the total population. Stratified analyses by gender and smoking status are presented in Table 4. For the total population we found associations for all the respiratory symptoms with the unadjusted model, which attenuated in the adjusted model. Still, moderate or heavy traffic was statistically significant for all symptoms, and little traffic was statistically significant for *nocturnal dyspnoea* and *nocturnal chest tightness*. Stratified analysis shows significant associations between self-reported traffic outside bedroom window and each respiratory symptom, primarily among females. *Nocturnal coughing*, *dyspnoea* and *chest tightness* were significantly associated with reporting traffic outside the bedroom window among female never- and occasional smokers. Furthermore, among female daily smokers, associations with *nocturnal dyspnoea* were seen, and associations with *nocturnal chest tightness* were found among female previous smokers. *Asthma attacks* was significantly associated with *moderate or heavy traffic (MHT) outside bedroom window* only among female never-smokers, whereas no associations were seen with *wheezing*. Female never-smokers that reported MHT were also more likely to report *nocturnal coughing* and *nocturnal dyspnoea* than never-smokers reporting no or little traffic outside their bedroom windows. The only statistically significant associations between reported exposure and respiratory symptoms in males were found among never-smoking men for *nocturnal dyspnoea* and among daily smoking men for *nocturnal chest tightness*.

**Table 4:** Unadjusted and adjusted odds ratios and 95% confidence intervals for the associations between self-reported traffic outside bedroom window and 5 different respiratory symptoms for the respondents in the Telemark study.

Total n = 16099		Traffic outside bedroom window	Respiratory symptoms last 12 months				
			Asthma attack	Nocturnal coughing	Nocturnal dyspnoea	Nocturnal chest tightness	Wheezing
Crude OR		No	1	1	1	1	1
		LT	1,16 (0,98–1,38)	**1,16 (1,07–1,26)**	**1,29 (1,14–1,47)**	**1,15 (1,04–1,26)**	**1,11 (1,02–1,20)**
		MHT	**1,75 (1,39–2,21)**	**1,60 (1,42–1,79)**	**1,97 (1,65–2,35)**	**1,72 (1,50–1,98)**	**1,47 (1,29–1,66)**
ORadj*, total population		No	1	1	1	1	1
		LT	1,06 (0,87–1,30)	1,09 (0,99–1,19)	**1,27 (1,09–1,47)**	**1,14 (1,02–1,28)**	1,10 (0,99–1,21)
		MHT	**1,35 (1,03–1,78)**	**1,28 (1,11–1,47)**	**1,60 (1,30–1,98)**	**1,44 (1,23–1,70)**	**1,24 (1,07–1,44)**
ORadj**, Never smokers	Male	No	1	1	1	1	1
		LT	1,35 (0,85–2,13)	0,99 (0,80–1,23)	**1,43 (1,01–2,03)**	1,16 (0,90–1,51)	1,22 (0,98–1,51)
		MHT	0,91 (0,38–2.19)	1,26 (0,90–1,79)	1,37 (0,78–2,13)	1,23 (0,80–1,90)	1,25 (0,86–1,81)
	Female	No	1	1	1	1	1
		LT	1,00 (0,71–1,41)	1,12 (0,95–1,31)	0,99 (0,74–1,32)	1,05 (0,85–1,29)	1,05 (0,87–1,26)
		MHT	**1,58 (1,02–2,43)**	**1,28 (1,01–1,64)**	**1,53 (1,03–2,26)**	1,33 (0,99–1,80)	1,08 (0,81–1,43)
ORadj**, Previous smokers	Male	No	1	1	1	1	1
		LT	0,52 (0,22–1,24)	0,93 (0,65–1,35)	1,20 (0,73–1,98)	0,83 (0,56–1,23)	0,95 (0,68–1,33)
		MHT	n<5	1,36 (0,79–2,35)	1,23 (0,56–2,71)	1,45 (0,83–2,52)	1,42 (0,85–2,36)
	Female	No	1	1	1	1	1
		LT	1,01 (0,58–1,76)	1,01 (0,78–1,31)	1,10 (0,71–1,71)	**1,49 (1,09–2,02)**	1,16 (0,87–1,57)
		MHT	1,83 (0,88–3,82)	1,25 (0,82–1,90)	1,47 (0,78–2,79)	**1,98 (1,24–3,15)**	1,49 (0,95–2,34)
ORadj**, Occational smokers	Male	No		1	1	1	1
		LT	n<5	1,04 (0,63–1,69)	0,98 (0,50–1,90)	0,69 (0,40–1,19)	0,86 (0,53–1,38)
		MHT	n<5	1,39 (0,66–2,91)	1,85 (0,76–4,52)	1,38 (0,64–2,98)	1,51 (0,74–3,09)
	Female	No	1	1	1	1	1
		LT	1,26 (0,44–3,60)	1,34 (0,90–1,99)	**2,39 (1,18–4,84)**	1,60 (0,98–2,63)	1,22 (0,78–1,90)
		MHT	2,51 (0,59–10,8)	**2,37 (1,29–4,34)**	2,34 (0,85–6,46)	2,00 (0,96–4,19)	1,79 (0,91–3,51)
ORadj**, Daily smokers	Male	No	1	1	1	1	1
		LT	1,04 (0,41–2,61)	1,01 (0,72–1,42)	1,05 (0,63–1,77)	1,08 (0,72–1,52)	0,83 (0,60–1,16)
		MHT	n<5	1,33 (0,80–2,22)	1,97 (0,98–3,94)	**1,76 (1,00–3,10)**	1,14 (0,69–1,88)
	Female	No	1	1	1	1	1
		LT	1,64 (0,96–2,81)	1,23 (0,93–1,64)	**2,16 (1,36–2,43)**	1,39 (0,99–1,95)	1,29 (0,96–1,74)
		MHT	1,25 (0,61–2,56)	1,01 (0,68–1,51)	**2,03 (1,12–3,67)**	1,31 (0,84–2,06)	1,16 (0,77–1,74)

* Adjusted for gender, age, marital status, educational level, employment status, living in Grenland area, type of housing, dampness/mould or water damage in house in the last 10 years, second-hand smoking, smoking status (never, previous, occasional, daily), number of pack-years, number of cigarettes per day, dust/gas exposure at work, and the other TRAP exposure

** Adjusted for age, marital status, educational level, employment status, living in Grenland area, type of housing, dampness/mould or water damage in house in the last 10 years, second-hand smoking, number of pack-years, number of cigarettes per day, dust/gas exposure at work, and the other TRAP exposure

The association between *time spent by foot daily along a moderate to heavy trafficked road* and single respiratory symptoms remained statistically significant for *nocturnal dyspnoea* and *wheezing* in the adjusted model (Table 5). In the stratified analyses, the associations with *nocturnal dyspnoea*, *nocturnal chest tightness* and *wheezing* were statistically significant among male daily smokers. Positive associations of similar magnitude, but not statistically significant, were found for *nocturnal chest tightness* and *wheezing* among male occasional smokers and for *wheezing* among male previous smokers. The only significant association between *time spent by foot daily along busy roads* and respiratory symptoms among women was among daily smoking females for *wheezing (Table 5)*

**Table 5:** Unadjusted and adjusted odds ratios and 95% confidence intervals for the associations between self-reported estimates of time spent by foot daily along moderate to heavy trafficked road and 5 different respiratory symptoms for the respondents in the Telemark study.

Time spent daily by foot along a busy road		Asthma attack	Nocturnal coughing	Nocturnal dyspnoea	Nocturnal chest tightness	Wheezing
Crude OR		**1,09 (1,01–1,19)**	**1,07 (1,02–1,12)**	**1,13 (1,06–1,20)**	**1,07 (1,01–1,22)**	**1,14 (1,09–1,19)**
ORadj*, total population		1,05 (0,95–1,16)	1,04 (0,99–1,09)	**1,08 (1,01–1,16)**	1,02 (0,96–1,08)	**1,09 (1,04–1,15)**
ORadj**, Never smokers	Male	1,02 (0,81–1,29)	0,99 (0,88–1,10)	1,12 (0,98–1,29)	1,02 (0,90–1,16)	1,02 (0,91–1,13)
	Female	0,97 (0,78–1,21)	1,03 (0,92–1,16)	1,01 (0,84–1,25)	1,03 (0,90–1,19)	1,02 (0,90–1,17)
ORadj**, Previous smokers	Male	1,11 (0,88–1,40)	1,05 (0,92–1,20)	1,01 (0,84–1,21)	0,83 (0,68–1,03)	1,10 (0,99–1,23)
	Female	1,17 (0,89–1,54)	1,04 (0,87–1,24)	1,09 (0,85–1,39)	1,03 (0,84–1,26)	1,02 (0,84–1,24)
ORadj**, Occasional smokers	Male	0,92 (0,48–1,73)	1,05 (0,86–1,28)	1,08 (0,85–1,37)	1,16 (0,96–1,40)	1,15 (0,96–1,37)
	Female	1,50 (0,52–4,28)	1,01 (0,66–1,55)	1,35 (0,71–2,58)	0,94 (0,55–1,63)	1,35 (0,87–2,11)
ORadj**, Daily smokers	Male	1,06 (0,79–1,72)	1,06 (0,94–1,19)	**1,20 (1,05–1,38)**	**1,13 (1,00–1,28)**	**1,14 (1,02–1,29)**
	Female	0,97 (0,71–1,31)	1,13 (0,95–1,34)	0,86 (0,64–1,16)	0,85 (0,68–1,06)	**1,20 (1,00–1,44)**

*Adjusted for gender, age, marital status, educational level, employment status, living in Grenland, type of housing, dampness/mould or water damage in house in the last 10 years, second-hand smoking, smoking status, number of pack-years, number of cigarettes per day, dust/gas exposure at work, and the other TRAP exposure

**Adjusted for age, marital status, educational level, employment status, living in Grenland, type of housing, dampness/mould or water damage in house in the last 10 years, second-hand smoking, number of pack-years, number of cigarettes per day, dust/gas exposure at work,and the other TRAP exposure

Results for total population and strata by gender and smoking status for 1-hour increase in exposure.

## Discussion

In this large, population-based study of adults from Telemark County in Norway, we have found statistically significant associations between reported *traffic outside bedroom window*, *time spent by foot daily along moderate to heavy trafficked road* and respiratory symptoms. The current findings are generally in line with previous studies linking residential TRAP exposure to increased risk for respiratory symptoms in persons living closer than 50 meters from a heavily trafficked road [9–11, 22, 46]. The associations between TRAP estimates and reported respiratory symptoms differed between men and women and varied by smoking status. Associations between *traffic outside the bedroom window* and respiratory symptoms were most prominent among women, with the strongest associations among occasional female smokers. Statistically significant associations between *time spent by foot daily along moderate to heavy trafficked road* and respiratory symptoms were revealed among daily smokers, primarily men.

The prevalence of reported respiratory symptoms was higher in females than in males (Table 2), and the association between *amount of traffic outside the bedroom window* and respiratory symptoms was stronger in women compared to men (Tables 3 and 4). Jedrychowski hypothesized that larger impact of air pollution on respiratory health in women may be caused by more time spent indoors at home, leading to more accurate assignment of residential air pollution exposure[47]. Whether this was the case in the population in the Telemark study is not clear. Normally, more time spent at home is related to whether you have a job or not, and in many countries more females than males stay at home. In the Telemark study, however, the proportion of male and female responders reporting being unemployed the last year is similar (16% vs 18%, respectively) (Table 1). Nevertheless, the finding in this study that symptom prevalence and association with TRAP exposure was higher in women may also indicate greater vulnerability to TRAP among females. Similar results have previously been described [12, 48]. Whether this vulnerability is physiological and reflects higher susceptibility towards respiratory morbidity in women, or if it reflects a more sensitive perception of traffic and respiratory symptoms, is not known. However, slightly greater airway reactivity in women has been shown[49]. A study of onset of asthma in adults living in Swedish cities used calculated TRAP estimates based on dispersion models as an exposure measure, and it found that females developed asthma more frequently than males [9]. Accordingly, our results showed a significant association between TRAP and asthma attacks only among never-smoking females reporting *moderate or heavy traffic outside bedroom window*. Also in the Atherosclerosis Risk in Communities study (ARIC), the risk of modest reduction in lung function was related to objective exposure estimates only in women[12]. These findings indicate that there are gender differences in respiratory vulnerability that are unrelated to individual perception of traffic or symptoms. On the other hand, annoyance from air pollution has previously been shown to be associated with factors such as reporting respiratory symptoms[50], female gender and high education[51]. Furthermore, women are found to report more frequent and severe symptoms than men in clinical settings and in health surveys[52, 53]. Also, Jacquemin and colleagues suggested that the level of annoyance could be a measure of perceived air quality, and that females with more symptoms may perceive air pollution more negatively than men, and consequently report higher amounts of traffic. Similar association between reported respiratory symptoms and level of annoyance were found in the SAPALDIA study[50] with stronger effects on respiratory morbidity from residential TRAP in male non-smokers than other subgroups[22]. In our study, significant associations between *traffic outside bedroom window* and respiratory symptoms were found for *nocturnal dyspnoea* among non-smoking males. In the study by Cesaroni and colleagues in Rome, Italy, an increased risk of asthma with higher self-reported traffic density was found, similar to our study, but the objective TRAP measures in that study could not confirm the observation even though the self-reported traffic estimate in general was correlated with objective measures of TRAP. Reporting bias or more sensitive perception of TRAP by persons with asthma were suggested explanations for this observation by the authors[13]. Given our cross-sectional design using self-reported exposures and outcomes, we cannot rule out bias in the reporting of respiratory symptoms and/or traffic estimates as possible explanations for the results. In studies such as ours, in which the association between subjectively measured variables is studied, there is a risk for common method bias. In such cases, the responder’s reporting will systematically affect the associations under investigation through, for instance, the personal style of reporting for each responder. This type of bias may generate associations between variables, which do not actually exist[54]. The response rate of the Telemark-study from which the present study population derives, was relatively low (33%). Assessment of non-response showed a somewhat higher prevalence of chronic cough and use of asthma medication among the participants compared with non-responders[44]. However, the prevalence of other respiratory symptoms and of physician-diagnosed asthma was similar between participants and non-responders indicating valid estimates. Nevertheless, the current results may not be entirely representative of the general population. Furthermore, although our data are cross-sectional, they are from a large sample of the general population and include a broad range of age categories. Importantly, and as is the case with all cross-sectional studies, no causal inferences can be drawn. This study was also limited in terms of geographic area; hence, additional studies are needed to confirm our findings.

A significant increase in *number of respiratory symptoms* was associated with increased *time spent by foot daily along a busy road* for male smokers. However, the model indicated that a noticeable increase in *number of respiratory symptoms* would be expected only after spending many hours daily by foot along busy roads. Hence, the general risk of experiencing respiratory symptoms associated with spending time by foot along a busy road was modest. Separating the data by each specific symptom and stratifying by gender and smoking status still singled out male smokers as the most sensitive group (Table 5). Even though this finding is in contradiction to what was observed for the risk associated with *traffic amount outside the bedroom window*, it is in accordance with findings from previous studies that have identified stronger respiratory effects from traffic exposure in males[10, 22]. However, twice as many men (n = 216) than women (n = 108) reported spending more than 2 hours daily by foot along a busy road. This may have contributed to the observed gender difference. Furthermore, reasons for spending many hours by foot along busy roads daily were not identified. Some individuals were possibly very physically active, commuting to work by foot, possibly exercising during the afternoons, and therefore spent considerable time along moderate or busy roads. Others may have had jobs where they spent time by foot along busy roads during the working hours, which may be more relevant for males than females. The respiratory status of the respondents would probably vary according to the rationale behind spending many hours daily by foot along busy roads. These possible confounding factors have not been accounted for in the present study.

In general, no consistent pattern of a difference between non-smokers and smokers was found when considering significant associations between *reported traffic amount outside bedroom window* and respiratory symptoms. The impact of smoking status varied between the symptoms (Tables 3 and 4). Significant associations between *time spent daily by foot along busy roads* and respiratory symptoms, both considering *number of respiratory symptoms* and separate symptoms, were, however, only found among smokers. These findings are partly inconsistent with previous studies in which non-smokers have been identified as the most sensitive group[10, 13, 22]. In fact, Künzli and colleagues [28] chose to only include non-smokers in their analyses based on the hypotheses that the respiratory exposure from cigarette smoking exceeds the pollutant concentration from all possible environmental pollution sources, and the smokers will therefore suffer effects mainly from their cigarette smoking. In our study, TRAP exposure related to activities outdoors close to roads was only significantly associated with respiratory symptoms among those who smoked every day, particularly men, suggesting that daily smoking men are the most sensitive group in this regard. It is also possible that this finding suggests a cumulative effect of smoking and TRAP in males. However, smoking status seemed to have little effect on associations between residential TRAP and respiratory symptoms, where women appeared to be more sensitive regardless of smoking status. Nevertheless, the associations between residential TRAP and respiratory symptoms in female occasional smokers were found to be stronger than the associations between TRAP exposure and the symptoms found for female daily smokers. It is known that all smoking, including occasional smoking, increases morbidity risk, and a dose-response pattern has been shown[55]. The total exposure burden for daily smokers is probably dominated by smoking and the associations between TRAP exposure, and respiratory symptoms may therefore be less clear than what is found among the female occasional smokers, who are expected to be less affected by smoking due to the lower dose.

A clear relationship between asthma and socioeconomic status has not been established, but an extra burden of asthma in populations with low socioeconomic status has been reported in previous studies[56,57]. Furthermore, living in close proximity to busy roads is often considered as less attractive housing, and may be associated with lower socioeconomic status as well[58]. Also, persons with asthma living in poverty have been shown to suffer worse effects of traffic exposure[14]. Socioeconomic status may therefore be an important confounding factor when investigating the association between living or walking close to busy roads and respiratory symptoms. In our analyses, we adjusted for educational level, marital status, employment status, type of housing, second hand smoking, personal smoking status and presence of mould and damp damage in the home to account for the influence of socioeconomic status on the estimated associations. These variables are possibly not adjusting our estimates sufficiently and may have resulted in some residual confounding. For example, income was not included in the first wave of the Telemark study. However, the economic contrasts in the Norwegian population are small, with a difference in income ratio of 2.8 between the 90^th^ decile and the 10^th^ decile in 2016[59]. The effect of income was therefore expected to be modest. Furthermore, we cannot exclude the possibility that including several adjustment factors may have resulted in diluted associations due to over-adjustment.

According to monitoring data from the WHO, there is substantial variability in the urban air pollution in the different regions of the world[60]. According to these monitoring data, the levels of PM in Scandinavia are generally lower than in other European countries, with annual mean PM concentrations below or close to the WHO guideline threshold values[61]. Nevertheless, TRAP is an issue of concern also in urban areas in Scandinavia and significant associations between TRAP exposure estimates and respiratory symptoms were found in the current study. According to these findings, the proportion of the population that experienced respiratory symptoms related to TRAP was limited to the 10% of the respondents that reported having their bedroom windows near a moderate or heavily trafficked road and smokers that spent several hours daily by foot along busy roads. In other words, these are the supposedly most exposed individuals in the current population. Consequently, for most of the population in the Telemark study, TRAP exposure was not associated with increased risk of experiencing respiratory symptoms, probably due to low levels of TRAP exposure. This finding indicates that the most trafficked areas in Telemark County may be close to a threshold for TRAP-induced respiratory symptoms. Other large cities in Norway, such as Oslo[31], have similar or higher AADT counts compared to the busiest roads in Grenland. However, Oftedal and colleagues could not find associations between TRAP exposure and respiratory illness in schoolchildren In Oslo[62]. This indicates that the TRAP levels in Norway’s largest city, even though they exceed national air quality thresholds annually, may be associated with lower respiratory risk compared to other European cities. The current findings do, nevertheless, indicate that the moderate TRAP levels found in Grenland may be associated with an increase in respiratory symptoms for the most exposed and susceptible individuals. This is in accordance with the findings of Modig[9, 27] and Andersen[6], who found respiratory outcomes in Swedish and Danish urban populations associated with TRAP levels comparable to those measured in the most polluted measurement stations in Grenland[40].

The general pattern was that the incidence rate ratios (IRR) were higher for the persons reporting *medium or heavy traffic outside their bedroom window* than those reporting *no* or *low amounts of traffic*, and most statistically significant associations between *traffic outside bedroom window* and symptoms were found in the medium- or high-traffic group. These findings imply a possible exposure-response relationship between TRAP and respiratory symptoms. This assumption is further supported by the statistically significant positive association between *time spent by foot along a busy road* and *number of respiratory symptoms*. Similarly, the SAPALDIA study observed a dose-response relationship between reported breathlessness and distance to the nearest main street and the length of main street segments around the home[22]. However, one major limitation in our study was that TRAP exposure estimates were self-reported. No objective exposure assessments or measurements of actual TRAP exposure such as individual PM or NOx levels were available, as this was beyond the scope of the Telemark study.

TRAP exposure in urban environments varies. Previous studies have shown that the exposure close to busy roads is 5–10 times higher compared to locations 50–200 m further away[63–66]. Hence, staying close to busy roads is associated with increased risk of TRAP exposure. The TRAP exposure estimate used in the present study—“*How much time daily do you spend by foot along a moderate to heavy trafficked road*?”—was therefore a reasonable estimate of an individual’s exposure to TRAP. Also, most TRAPs, such as NOx and ultrafine PM, penetrates indoors. Most Norwegian houses do not have an air-condition system, but are naturally ventilated through simple ventilation hatches or just by opening windows. Furthermore, most people are sleeping with an open bedroom window in Norway[67]. Hence, TRAP will easily find its way inside from the surroundings. In the Nordic RHINE study, an indoor/outdoor NO_2_ ratio of 0.4–0.7 was found[68]. This implies that living close to a busy road affects even the indoor climate considerably, and most persons spend many hours daily in their bedrooms. Even though traffic amount along most roads is low during the night, TRAP that penetrates indoors is not expected to clear rapidly but to accumulate during the day, and the TRAP levels inside will reflect an average daily TRAP exposure level. Hence, using the position of the bedroom window relative to a trafficked road with a traffic estimate was considered a reasonable exposure estimate for residential TRAP exposure.

Both exposure measures were proxies for near-road TRAP exposure and were based on subjective estimates of what a ‘little’, ‘moderate’ or ‘high’ traffic road was. These terms are relative since each person’s subjective estimate of traffic amount will differ and probably be influenced by whether the respondent lived in a rural or urban area. Self-reported traffic estimates have been shown to be less accurate in rural areas than urban areas [69], an effect we cannot totally rule out in our data. Whether or not the respondents lived in the urban Grenland area was, however, included in the analyses in order to adjust for this possible effect. Also, caution should be made when comparing the findings in the current study to other populations, given the subjective nature of the traffic estimate and the fact that even the most busy roads in the most densely populated areas of Telemark are merely moderately trafficked in a global, urban context [30, 33–36].

Various exposure estimation strategies have been applied in order to investigate the impact of residential TRAP on respiratory morbidity. Objective exposure measures have been obtained by identifying the home address for each study subject and thereafter calculating individual TRAP exposure using, for instance, geographic information system (GIS) data, dispersion models including emission data and air quality measurements, traffic counts or estimation of residential proximity to busy roads[6, 9, 10, 12, 14, 22, 25, 26, 28, 62]. Still, self-reported traffic estimates are commonly applied [11, 13, 21, 24, 70]. Self-reported traffic estimates are cost-efficient and easily obtained but undeniably subjective perceptions prone to various biases, particularly when traffic estimates and health are subjects to the same questionnaire, and this may lead to misclassification. This has particularly been seen when parents of respiratory symptomatic children report traffic exposure outside their home[71], which is often classified as recall bias. Quantitative exposure estimates such as air quality measurements and dispersion modelling are more objective and less likely to be biased, yet are also resource-demanding and complex methods. Self-reported estimations of traffic, such as traffic amount or annoyance from traffic, compared to objective quantitative exposure measures have, however, shown that subjective assessments are reliable in homogeneous communities[13, 50, 72], particularly in urban areas[69]. Oglesby and colleagues concluded that reported annoyance is a function of true exposure, although it is distorted by subjective factors[50]. However, future work on the Telemark study would benefit from including objective modelling to assess the TRAP exposure for each responder. Two major studies are currently investigating this topic: the ELAPSE study[73, 74] and the NordicWelfAir study[75], where Norway is included. These will hopefully provide valuable insight into this topic in the future.

## Conclusion

Overall, we found statistically significant associations between self-reported TRAP exposure and respiratory symptoms in a large, random sample from a population living in an area with modest TRAP levels. The results of this population-based study suggest that primarily women, both never-smokers and smokers, are at risk for experiencing respiratory symptoms if they have their bedroom window close to what in Telemark County is considered a moderate to heavy trafficked road. Additionally, our findings suggest that people, particularly male smokers, that spend many hours by foot daily along a busy road suffer an increased risk of respiratory symptoms. Furthermore, the results provide a basis for future studies where objective exposure measures should be included in order to explore the currently revealed relationship between modest TRAP levels and respiratory symptoms.

## Supporting information

S1 Table Correlation between reported symptoms. Correlation between reported symptoms the responders had experienced during the last 12 months; Asthma attack, wheezing, nocturnal dyspnoea, nocturnal cough, chest tightness. There were fair to moderate significant (p<0,05) correlations (Spearman) between all symptoms reported by the responders.
(DOCX)

## References

[1] Guarnieri M, Balmes JR. Outdoor air pollution and asthma. The Lancet. 2014;383(9928):1581–92. 10.1016/S0140-6736(14)60617-6 PMC4465283 24792855

[2] Brauer M, Amann M, Burnett RT, Cohen A, Dentener F, Ezzati M, et al Exposure assessment for estimation of the global burden of disease attributable to outdoor air pollution. Environmental science & technology. 2012;46(2):652–60. 22148428 10.1021/es2025752 PMC4043337

[3] Perez L, Declercq C, Iñiguez C, Aguilera I, Badaloni C, Ballester F, et al Chronic burden of near-roadway traffic pollution in 10 European cities (APHEKOM network). European Respiratory Journal. 2013;42(3):594–605. 10.1183/09031936.00031112 23520318

[4] Krzyzanowski Michal, Schneider BK-DaJ. Health effects of transport-related air pollution. The Regional Office for Europe of the World Health Organization 2005 2005. Contract No.: ISBN 92 890 1373 7.

[5] Laumbach RJ, Kipen HM. Respiratory health effects of air pollution: Update on biomass smoke and traffic pollution. Journal of Allergy and Clinical Immunology. 2012;129(1):3–11. 10.1016/j.jaci.2011.11.021 22196520 PMC3272333

[6] Andersen ZJ, Hvidberg M, Jensen SS, Ketzel M, Loft S, Sørensen M, et al Chronic Obstructive Pulmonary Disease and Long-Term Exposure to Traffic-related Air Pollution. American Journal of Respiratory and Critical Care Medicine. 2011;183(4):455–61. 10.1164/rccm.201006-0937OC 20870755

[7] Health_Effects_Institute. Traffic related air-pollution: acritical review of the litterature on emissions, exposure, and health effects. Pollution. PotHEoT-RA; 2010.

[8] Pope IC, Burnett RT, Thun MJ, et al Lung cancer, cardiopulmonary mortality, and long-term exposure to fine particulate air pollution. JAMA. 2002;287(9):1132–41. 10.1001/jama.287.9.1132 11879110 PMC4037163

[9] Modig L, Torén K, Janson C, Jarvholm B, Forsberg B. Vehicle exhaust outside the home and onset of asthma among adults. European Respiratory Journal. 2009;33(6):1261–7. 10.1183/09031936.00101108 19251785

[10] Garshick E, Laden F, Hart JE, Caron A. Residence Near a Major Road and Respiratory Symptoms in U.S. Veterans. Epidemiology (Cambridge, Mass). 2003;14(6):728–36. 10.1097/01.ede.0000082045.50073.66 PMC1351073 14569190

[11] Heinrich J, Topp R, Gehring U, Thefeld W. Traffic at residential address, respiratory health, and atopy in adults: the National German Health Survey 1998. Environmental Research. 2005;98(2):240–9. 10.1016/j.envres.2004.08.004 15820731

[12] Kan H, Heiss G, Rose KM, Whitsel E, Lurmann F, London SJ. Traffic exposure and lung function in adults: the Atherosclerosis Risk in Communities study. Thorax. 2007;62(10):873–9. 10.1136/thx.2006.073015 17442705 PMC2094260

[13] Cesaroni G, Badaloni C, Porta D, Forastiere F, Perucci CA. Comparison between various indices of exposure to traffic-related air pollution and their impact on respiratory health in adults. Occupational and Environmental Medicine. 2008;65(10):683–90. 10.1136/oem.2007.037846 18203803 PMC2771851

[14] Meng Y-Y, Wilhelm M, Rull RP, English P, Nathan S, Ritz B. Are Frequent Asthma Symptoms Among Low-Income Individuals Related to Heavy Traffic Near Homes, Vulnerabilities, or Both? Annals of Epidemiology. 2008;18(5):343–50. 10.1016/j.annepidem.2008.01.006 18433665

[15] Oftedal B, Brunekreef B, Nystad W, Madsen C, Walker S-E, Nafstad P. Residential Outdoor Air Pollution and Lung Function in Schoolchildren. Epidemiology. 2008;19(1):129–37. 10.1097/EDE.0b013e31815c0827 18091005

[16] Janssen NAH, Van Vliet PHN, Aarts F, Harssema H, Brunekreef B. Assessment of exposure to traffic related air pollution of children attending schools near motorways. Atmospheric Environment. 2001;35.

[17] van Vliet P, Knape M, de Hartog J, Janssen N, Harssema H, Brunekreef B. Motor Vehicle Exhaust and Chronic Respiratory Symptoms in Children Living near Freeways. Environmental Research. 1997;74(2):122–32. 10.1006/enrs.1997.3757 9339225

[18] Duhme H, Weiland SK, Keil U, Kraemer B, Schmid M, Stender M, et al The Association between Self-Reported Symptoms of Asthma and Allergic Rhinitis and Self-Reported Traffic Density on Street of Residence in Adolescents. Epidemiology. 1996;7(6):578–82. 10.1097/00001648-199611000-00003 8899382

[19] Weiland SK, Mundt KA, Rückmann A, Keil U. Self-reported wheezing and allergic rhinitis in children and traffic density on street of residence. Annals of Epidemiology. 1994;4(3):243–7. 10.1016/1047-2797(94)90103-1 7519948

[20] Delfino RJ, Wu J, Tjoa T, Gullesserian SK, Nickerson B, Gillen DL. Asthma Morbidity and Ambient Air Pollution: Effect Modification by Residential Traffic-Related Air Pollution. Epidemiology. 2014;25(1):48–57. 10.1097/EDE.0000000000000016 24240657

[21] Skrzypek M, Kowalska M, Kasznia-Kocot J, Czech EM, Niewiadomska E. Respiratory health problems in adolescents living near main roads in the Upper Silesian industrial zone, Poland. International Journal of Occupational Medicine and Environmental Health. 2019;32(4):553–67. 10.13075/ijomeh.1896.01342 31303647

[22] Bayer-Oglesby L, Schindler C, Hazenkamp-von Arx ME, Braun-Fahrländer C, Keidel D, Rapp R, et al Living near Main Streets and Respiratory Symptoms in AdultsThe Swiss Cohort Study on Air Pollution and Lung Diseases in Adults. American Journal of Epidemiology. 2006;164(12):1190–8. 10.1093/aje/kwj338 17032694

[23] Nitta H, Sato T, Nakai S, Maeda K, Aoki S, Ono M. Respiratory health associated with exposure to automobile exhaust. I. Results of cross-sectional studies in 1979, 1982, and 1983. Archives of Environmental Health. 1993;48. 10.1080/00039896.1993.9938393 7680850

[24] Brunekreef B, Stewart AW, Anderson HR, Lai CKW, Strachan DP, Pearce N, et al Self-Reported Truck Traffic on the Street of Residence and Symptoms of Asthma and Allergic Disease: A Global Relationship in ISAAC Phase 3. Environmental Health Perspectives. 2009;117(11):1791–8. 10.1289/ehp.0800467 20049134 PMC2801184

[25] Cook AG, deVos AJ, Pereira G, Jardine A, Weinstein P. Use of a total traffic count metric to investigate the impact of roadways on asthma severity: a case-control study. Environmental Health. 2011;10(1):52. 21631953 10.1186/1476-069X-10-52 PMC3127974

[26] Atkinson R, Carey IM, Kent AJ, Van Staa T, Anderson H, Cook DG. Long-term exposure to outdoor air pollution and the incidence of chronic obstructive pulmonary disease in a national English cohort. Occup Environ Med. 2014:oemed-2014-102266. 10.1136/oemed-2014-102266 PMC4283678 25146191

[27] Modig L, Jarvholm B, Ronnmark E, Nystrom L, Lundback B, Andersson C, et al Vehicle exhaust exposure in an incident case-control study of adult asthma. Eur Respir J. 2006;28. 10.1183/09031936.06.00071505 16540504

[28] Künzli N, Bridevaux P-O, Liu L-JS, Garcia-Esteban R, Schindler C, Gerbase MW, et al Traffic-related air pollution correlates with adult-onset asthma among never-smokers. Thorax. 2009;64(8):664–70. 10.1136/thx.2008.110031 19359271

[29] Anderson HR, Favarato G, Atkinson RW. Long-term exposure to air pollution and the incidence of asthma: meta-analysis of cohort studies. Air Quality, Atmosphere & Health. 2013;6(1):47–56.

[30] Zhu Y, Hinds WC, Kim S, Sioutas C. Concentration and Size Distribution of Ultrafine Particles Near a Major Highway. Journal of the Air & Waste Management Association. 2002;52(9):1032–42. 12269664 10.1080/10473289.2002.10470842

[31] NVDB NNRD. Vegkart.no http://www.vegdata.no/vegkart/: Norwegian Road Authorities; 2018 [updated June 18th 2018).

[32] StatisticsNorway. https://www.ssb.no/kommunefakta [updated March 2018; cited 2018 June 18th].

[33] administration St. Se Sveriges vägar på karta [Web page]. https://nvdb2012.trafikverket.se/SeTransportnatverket2018 [Available from: https://nvdb2012.trafikverket.se/SeTransportnatverket.

[34] Average daily traffic volume (motor vehicles)-2015 [Internet]. 2016 [cited 30.04.2018].

[35] Cesaroni G, Badaloni C, Romano V, Donato E, Perucci CA, Forastiere F. Socioeconomic position and health status of people who live near busy roads: the Rome Longitudinal Study (RoLS). Environmental Health. 2010;9:41–. 10.1186/1476-069X-9-41 20663144 PMC2918588

[36] Brauer M, Hoek G, van Vliet P, Meliefste K, Fischer P, Gehring U, et al Estimating Long-Term Average Particulate Air Pollution Concentrations: Application of Traffic Indicators and Geographic Information Systems. Epidemiology. 2003;14(2):228–39. 10.1097/01.EDE.0000041910.49046.9B 12606891

[37] WHO WHOOaEHT. WHO Air quality guidelines for particulate matter, ozone, nitrogen dioxide and sulfur dioxide: global update 2005: summary of risk assessment. http://apps.who.int/iris/handle/10665/69477: IRIS WHO; 2006.

[38] FHI NNIoPh. Norwegian Air Quality Criteria https://www.fhi.no/nettpub/luftkvalitet/sammendrag/sammendrag/#oversikt-luftkvalitetskriterier2013 [updated 13. February 2018; cited 2018 June 19th]. Available from: https://www.fhi.no/nettpub/luftkvalitet/sammendrag/sammendrag/#oversikt-luftkvalitetskriterier.

[39] Bymiljøetaten OK. Luftkvaliteten i Oslo i 2017 –En oppsummering. http://luftkvalitet.info/Libraries/Rapporter/Luftkvaliteten_i_Oslo_i_2017_-_En_oppsummering.sflb.ashx: oslo Kommune Bymiljøetaten; 2018 16. March 2018.

[40] Bamble kommune Pk, Skien kommune, Industrien i Grenland og Statens vegvesen. Luftkvalitet.info. Målenettverket i Grenland. Årsrapport 2017. http://luftkvalitet.info/Libraries/Rapporter/Grenland_%c3%a5rsrapport_2017.sflb.ashx: Bamble kommune, Porsgrunn kommune, Skien kommune, Industrien i Grenland og Statens vegvesen; 2018 23. April 2018.

[41] WHO WHO. Global Ambient air Pollition Map. Annual mean PM 2.5 and PM 10 concentrations. http://maps.who.int/airpollution/: WHO; 2018 [updated 2018; cited 2019 24. October 2019]. Available from: http://maps.who.int/airpollution/.

[42] Abrahamsen R, Fell AKM, Svendsen MV, Andersson E, Torén K, Henneberger PK, et al Association of respiratory symptoms and asthma with occupational exposures: findings from a population-based cross-sectional survey in Telemark, Norway. BMJ Open. 2017;7(3). 10.1136/bmjopen-2016-014018 PMC5372104 28336744

[43] Fell A, Abrahamsen R, Henneberger P, Svendsen M, Andersson E, Torén K, et al Breath-taking jobs: a case–control study of respiratory work disability by occupation in Norway. Occup Environ Med. 2016:oemed-2015-103488. 10.1136/oemed-2015-103488 PMC5013093 27365181

[44] Abrahamsen R, Svendsen MV, Henneberger PK, Gundersen GF, Torén K, Kongerud J, et al Non-response in a cross-sectional study of respiratory health in Norway. BMJ Open. 2016;6(1). 10.1136/bmjopen-2015-009912 PMC4716229 26739738

[45] Torén K, Ekerljung L, Kim J-L, Hillström J, Wennergren G, Rönmark E, et al Adult-onset asthma in west Sweden – Incidence, sex differences and impact of occupational exposures. Respiratory Medicine.105(11):1622–8. 10.1016/j.rmed.2011.06.003 21757331

[46] Johannessen A, de Marco R, Skorge T, Waatevik M, Svanes Ø, Real F, et al Self-reported traffic air pollution and respiratory symptoms among never-smokers and ever-smokers in a general population. European Respiratory Journal. 2014;44(Suppl 58).

[47] Jedrychowski W, Krzyzanowski M. Ventilatory lung function and chronic chest symptoms among the inhabitants of urban areas with various levels of acid aerosols: prospective study in Cracow. Environmental Health Perspectives. 1989;79:101–7. 10.1289/ehp.8979101 2707189 PMC1567559

[48] Doiron D, de Hoogh K, Probst-Hensch N, Fortier I, Cai Y, De Matteis S, et al Air pollution, lung function and COPD: results from the population-based UK Biobank study. European Respiratory Journal. 2019;54(1):1802140 10.1183/13993003.02140-2018 31285306

[49] Yunginger JW, Reed CE, O'Connell EJ, Joseph Melton I L., O'Fallon WM, Silverstein MD. A Community-based Study of the Epidemiology of Asthma: Incidence Rates, 1964–1983. American Review of Respiratory Disease. 1992;146(4):888–94. 10.1164/ajrccm/146.4.888 1416415

[50] Oglesby L, Künzli N, Monn C, Schindler C, Ackermann-Liebrich U, Leuenberger P, et al Validity of Annoyance Scores for Estimation of Long Term Air Pollution Exposure in Epidemiologic Studies The Swiss Study on Air Pollution and Lung Diseases in Adults (SAPALDIA). American Journal of Epidemiology. 2000;152(1):75–83. 10.1093/aje/152.1.75 10901332

[51] Jacquemin B, Sunyer J, Forsberg B, Aguilera I, Briggs D, García-Esteban R, et al Home Outdoor NO2 and New Onset of Self-Reported Asthma in Adults. Epidemiology. 2009;20(1):119–26. 10.1097/EDE.0b013e3181886e76 18923331

[52] van Wijk CMTG, Kolk AM. Sex differences in physical symptoms: The contribution of symptom perception theory. Social Science & Medicine. 1997;45(2):231–46. 9225411 10.1016/s0277-9536(96)00340-1

[53] Barsky AJ, Peekna HM, Borus JF. Somatic Symptom Reporting in Women and Men. Journal of General Internal Medicine. 2001;16(4):266–75. 10.1046/j.1525-1497.2001.00229.x 11318929 PMC1495200

[54] Podsakoff PM, MacKenzie SB, Lee J-Y, Podsakoff NP. Common method biases in behavioral research: A critical review of the literature and recommended remedies. Journal of Applied Psychology. 2003;88(5):879–903. 10.1037/0021-9010.88.5.879 14516251

[55] Løchen M-L, Gram IT, Mannsverk J, Mathiesen EB, Njølstad I, Schirmer H, et al Association of occasional smoking with total mortality in the population-based Tromsø study, 2001–2015. BMJ open. 2017;7(12):e019107–e. 10.1136/bmjopen-2017-019107 29288187 PMC5770901

[56] Goodman DC, Stukel TA, Chang C-h. Trends in Pediatric Asthma Hospitalization Rates: Regional and Socioeconomic Differences. Pediatrics. 1998;101(2):208–13. 10.1542/peds.101.2.208 9445493

[57] Castro M, Schechtman KB, Halstead J, Bloomberg G. Risk Factors for Asthma Morbidity and Mortality in a Large Metropolitan City. Journal of Asthma. 2001;38(8):625–35. 10.1081/jas-100107540 11758891

[58] Gunier RB, Hertz A, von Behren J, Reynolds P. Traffic density in California: Socioeconomic and ethnic differences among potentially exposed children. Journal Of Exposure Analysis And Environmental Epidemiology. 2003;13:240 10.1038/sj.jea.7500276 12743618

[59] Statistics Norway Sn. Income differences https://www.ssb.no/inntekt-og-forbruk/statistikker/ifhus: www.ssb.no; 2018 [updated 13. december 2017; cited 2018 21. June ]. Available from: https://www.ssb.no/inntekt-og-forbruk/statistikker/ifhus.

[60] WHO WHO. World Health Organization: Global ambient air pollution interactive map [Web page]. http://maps.who.int/airpollution/2018 [cited 2018 30. April].

[61] WHO WHO. WHO Global Urban Ambient Air Pollution Database. In: Organization WH, editor. Update 2016 ed. http://www.who.int/phe/health_topics/outdoorair/databases/cities/en/2016.

[62] Oftedal B, Nystad W, Brunekreef B, Nafstad P. Long-Term Traffic-Related Exposures and Asthma Onset in Schoolchildren in Oslo, Norway. Environmental Health Perspectives. 2009;117(5):839–44. 10.1289/ehp.11491 19478970 PMC2685850

[63] Rodes CE, Holland DM. Variations of NO, NO2 and O3 concentrations downwind of a Los Angeles freeway. Atmospheric Environment (1967). 1981;15(3):243–50.

[64] Kuhler M, Kraft J, Koch W, Windt H. Dispersion of Car Emissions in the Vicinity of a Highway In: Grefen K, Löbel J, editors. Environmental Meteorology: Proceedings of an International Symposium held in Würzburg, FRG, 29 September– 1 October 1987. Dordrecht: Springer Netherlands; 1988 p. 39–47.

[65] Monn C, Carabias V, Junker M, Waeber R, Karrer M, Wanner HU. Small-scale spatial variability of particulate matter < 10 μm (PM10) and nitrogen dioxide. Atmospheric Environment. 1997;31(15):2243–7.

[66] Zhou Y, Levy JI. Factors influencing the spatial extent of mobile source air pollution impacts: a meta-analysis. BMC Public Health. 2007;7(1):89. 17519039 10.1186/1471-2458-7-89 PMC1890281

[67] Evandt J, Oftedal B, Hjertager Krog N, Nafstad P, Schwarze PE, Marit Aasvang G. A Population-Based Study on Nighttime Road Traffic Noise and Insomnia. Sleep. 2016;40(2). 10.1093/sleep/zsw055 28364487

[68] Hagenbjork-Gustafsson A, Forsberg B, Hestvik G, Karlsson D, Wahlberg S, Sandstrom T. Measurements of indoor and outdoor nitrogen dioxide concentrations using a diffusive sampler. Analyst. 1996;121(9):1261–4. 10.1039/an9962101261 8831283

[69] Heinrich J, Gehring U, Cyrys J, Brauer M, Hoek G, Fischer P, et al Exposure to traffic related air pollutants: self reported traffic intensity versus GIS modelled exposure. Occupational and Environmental Medicine. 2005;62(8):517–23. 10.1136/oem.2004.016766 16046603 PMC1741068

[70] Ciccone G, Forastiere F, Agabiti N, Biggeri A, Bisanti L, Chellini E, et al Road traffic and adverse respiratory effects in children. SIDRIA Collaborative Group. Occupational and Environmental Medicine. 1998;55(11):771–8. 10.1136/oem.55.11.771 9924455 PMC1757532

[71] Kuehni CE, Strippoli M-PF, Zwahlen M, Silverman M. Association between reported exposure to road traffic and respiratory symptoms in children: evidence of bias. International Journal of Epidemiology. 2006;35(3):779–86. 10.1093/ije/dyl022 16513809

[72] Forsberg B, Stjernberg N, Wall S. People can detect poor air quality well below guideline concentrations: a prevalence study of annoyance reactions and air pollution from traffic. Occupational and Environmental Medicine. 1997;54(1):44–8. 10.1136/oem.54.1.44 9072033 PMC1128634

[73] ELAPSE. http://www.elapseproject.eu/2019 [Available from: http://www.elapseproject.eu/.

[74] de Hoogh K, Chen J, Gulliver J, Hoffmann B, Hertel O, Ketzel M, et al Spatial PM2.5, NO2, O3 and BC models for Western Europe–Evaluation of spatiotemporal stability. Environment International. 2018;120:81–92. 10.1016/j.envint.2018.07.036 30075373

[75] NordicWelfAir. http://projects.au.dk/nordicwelfair/2019 [Available from: http://projects.au.dk/nordicwelfair/.

