# Supplementary material for: Self-reported traffic-related air pollution and respiratory symptoms among adults in an area with modest levels of traffic
Source: PLoS One. 2019 Dec 12;14(12):e0226221. doi: 10.1371/journal.pone.0226221 (PMC6907824; doi:10.1371/journal.pone.0226221)
Supplement: S1 Table — Correlation between reported symptoms the responders had experienced during the last 12 months; Asthma attack, wheezing, nocturnal dyspnoea, nocturnal cough, chest tightness. There were fair to moderate significant (p<0,05) correlations (Spearman) between all symptoms reported by the responders. (DOCX) [file pone.0226221.s001.docx]

**S1 Table**

|  | Asthma attack | Wheezing | Nocturnal dyspnoea | Nocturnal cough | Chest tightness |
| --- | --- | --- | --- | --- | --- |
| Asthma attack | **---------------------** | **r_s_= 0.33 p<0,00** | **r_s_= 0.35 p<0,00** | **r_s_= 0.19 p<0,00** | **r_s_= 0.32 p<0,00** |
| Wheezing | **r_s_= 0.33 p<0,00** | **-----------------------** | **r_s_= 0.32 p<0,00** | **r_s_= 0.34 p<0,00** | **r_s_= 0.48 p<0,00** |
| Nocturnal dyspnoea | **r_s_= 0.35 p<0,00** | **r_s_= 0.32 p<0,00** | **---------------------** | **r_s_= 0.26 p<0,00** | **r_s_= 0.52 p<0,00** |
| Nocturnal cough | **r_s_= 0.19 p<0,00** | **r_s_= 0.34 p<0,00** | **r_s_= 0.26 p<0,00** | **---------------------** | **r_s_= 0.35 p<0,00** |
| Chest tightness | **r_s_= 0.32 p<0,00** | **r_s_= 0.48 p<0,00** | **r_s_= 0.52 p<0,00** | **r_s_= 0.35 p<0,00** | ---------------------- |
